# Black Wattle (*Acacia mearnsii*) Condensed Tannins as Feed Additives to Lactating Dairy Cows

**DOI:** 10.3390/ani10040662

**Published:** 2020-04-11

**Authors:** Andre S. Avila, Maximiliane A. Zambom, Andressa Faccenda, Caroline H. Werle, Ana R. E. Almeida, Cibele R. Schneider, Dieisson G. Grunevald, Antonio P. Faciola

**Affiliations:** 1 Department of Animal Science, State University of Western Parana, Marechal Candido Rondon, PR 85960-000 Brazil; mazambom@hotmail.com (M.A.Z.); carol_qp90@hotmail.com (C.H.W.); anaruthestrela@gmail.com (A.R.E.A.); cibeleregina17@hotmail.com (C.R.S.); dieisson_dgg@hotmail.com (D.G.G.); 2 Department of Animal Science, State University of Maringa, Maringa, PR 87020-900, Brazil; andressafaccenda@hotmail.com; 3 Department of Animal Sciences, University of Florida, Gainesville, FL 32611, USA; afaciola@ufl.edu

**Keywords:** digestibility, intake, milk protein, polyphenols, secondary compounds

## Abstract

**Simple Summary:**

Plant compounds such as condensed tannins can be used to modulate ruminal fermentation, and to improve feed utilization and the final product quality in dairy cattle. In this study, we evaluated the inclusion of condensed tannins from Black Wattle (*Acacia mearnsii*) at levels up to 2% of the dry matter in the diets of dairy cows and its effects on feed intake, nutrient digestibility, milk production and composition. Condensed tannin inclusion in the diets did not affect feed or nutrient intake. Digestibility of diet dry matter and neutral detergent fiber was highest at inclusion levels of 1.22% and 1.14%, respectively. There was no effect of tannin inclusion on milk production; however, there was a reduction in milk casein concentration.

**Abstract:**

The objective of this study was to evaluate the effects of five levels of condensed tannins (CT) from black wattle (*Acacia mearnsii*) in the diets of lactating dairy cows on intake, nutrient digestibility, ruminal microbial protein synthesis, milk production, composition, oxidative profile, and blood metabolites. Five Holstein cows (88 ± 26.8 days in milk) were allocated in a 5 x 5 Latin square design for a period of 20 days (14 days of diet adaptation and six for sampling). Treatments were the inclusion levels of CT at 0, 5, 10, 15 and 20 g/kg of dry matter (DM) in the diet. There was no effect of CT on DM intake. The digestibility of DM and neutral detergent fiber changed quadratically, with the maximum values at 12.2 and 11.4 g/kg of DM, respectively. There was no effect on ruminal microbial protein synthesis and milk production; however, milk casein concentration was reduced linearly. There was no effect on the milk oxidative profile. Inclusion of CT at levels up to 20 g/kg of DM did not affect intake or microbial protein synthesis; however, added CT depressed the production of energy corrected milk and milk casein concentration.

## 1. Introduction

Condensed tannins are plant secondary metabolites that have high molecular weight and can be classified into two classes: condensed and hydrolyzed. Condensed tannins (CT) have the ability to form complexes mainly with proteins, polysaccharides, and minerals (Al, Ca, Co, Cu, Fe, Mg, Mn, P, and Zn) [1,2] and may reduce their bioavailability [3]; in addition, they may affect ruminal microorganisms [4].

Low to moderate concentrations of CT (2% to 4% of dry matter) in ruminant diets may increase the post-ruminal flow of non-ammonia nitrogen due to their ability to form reversible complexes with feed proteins [5], which promotes greater availability of amino acids in the small intestine [6]; however, it is also possible that part of the tannin–protein complexes have reduced digestibility in the abomasum [7] or that free tannins reaching the duodenum could inactivate intestinal enzymes or rebind to protein [8].

The formation of tannin–protein complexes depends on many factors such as tannin and protein chemistry (e.g., proline content), protein isoelectric point, pH, ionic strength of the solution and also the presence of other compounds. Tannins can react with a wide set of different organic N compounds including arginine (from all amino acids), nitrogen bases, polyamines, chitin, and chitosan [9,10].

The reduction in ruminal protein degradation rate may reduce ruminal ammonia concentrations and increase the escape of rumen undegraded protein (RUP), which may improve the efficiency of nitrogen utilization [11]. However, high concentrations of tannins can have detrimental effects on animal performance [1,12].

It is likely that CT may not be degraded and absorbed from the digestive tract, however, it may indirectly affect antioxidant status in animal tissues [13] and may improve some parameters of products quality such as milk and meat [14]. The optimum dietary tannin levels to improve animals’ performance and antioxidant capacity is still unknown. Among the different tannin sources available, the extract of CT from Black Wattle (*Acacia mearnsii*) has shown potential as a feed additive for ruminants [15]. However, the results are still inconsistent and depend on diet and feeding regime [13,16,17].

The objective of this study was to evaluate the effects of increasing levels of CT from black wattle (*Acacia mearnsii*) in the diets of lactating dairy cows on intake, nutrient digestibility, ruminal microbial protein synthesis, milk production, composition, oxidative profile, and blood metabolites. Our hypothesis was that moderate levels of black wattle CT could improve efficiency of milk production without negatively affecting feed intake and ruminal microbial protein synthesis but could have a positive effect on the milk oxidative profile.

## 2. Materials and Methods

This experiment was conducted at the experimental farm of the State University of Western Parana (Unioeste), Brazil, located at 24°31’55.3″ S, 54°01’08.0″ W and 392 m alt. The animal experiment protocol was approved by the Unioeste Animal Ethics Committee (number 54/16).

Five multiparous Holstein cows were used. Cows were 88 (±26.8) days in milk; initially, milk production averaged 31.4 (±4.14) kg and body weight was 608 (±72 kg). Cows were distributed in a 5 × 5 Latin square, balanced for carryover effects, with an experimental period of 20 days (14 for adaptation and six days for collection). Treatments were inclusion levels of extract of CT from black wattle (*Acacia mearnsii*) at 0, 6.1, 12.2, 18.4, and 24.6 g/kg of diet dry matter (DM) of diet, which was mixed with the other ingredients in the concentrate (Table 1). The extract was obtained in a very fine powder form, it was well mixed and there were no visual signs of sorting. According to the manufacturer, the product contained 80.52% of CT, 18.04% non-tannins, 1.44% insolubles, 2.11% ash, 59.80 ppm of iron, pH 4.9% and 5.65% humidity. Thus, considering the presumed tannin concentration in the commercial product, the doses chosen were 0, 5, 10, 15, and 20 g/kg CT in dietary DM (Table 2).

Animals were housed in a covered barn with tie-stall pens with individual feed and water troughs. Milking was performed twice a day and feed was offered after milking, with 70% of the total DM given at 06:00 and the remaining 30% at 16:00. The animals were weighed at the beginning and end of each period, before the morning feeding. Feed refusals were weighed for adjusting the amount of feed provided for each cow in order to ensure refusals between 5% and 10% of diet DM.

Intake was measured daily by weighing the feed provided and the refusals; feed intake was calculated for statistical analysis during the collection period (from the 15th to the 20th day) in which daily samples were collected and stored for further analysis. For digestibility evaluation, samples of feces (165 g) were collected directly from the rectum according to the following schedule: 15th day at 08:00, 16th day at 10:00, 17th day at 12:00, 18th day at 14:00, 19th day at 16:00 and 20th day at 18:00. On the 19th day, feces were collected a little earlier in relation to the other days in order not to coincide with the time of milking.

Subsequently the samples were oven dried (55 °C for 72 h) and ground through 1 mm sieves in a Willey type mill. Samples were pooled for feed, refusals and feces, resulting in one sample of each, per animal per period. Samples were analyzed according to AOAC [19] methodology for DM (DM, method 934.01), ether extract (EE, method 920.85), ash (method 938.08), crude protein (CP, method 981.10) and neutral detergent fiber (NDF) according to Van Soest et al. [20]. The organic matter (OM) was calculated by the difference between the ash content and total DM. Non-fibrous carbohydrates were calculated by the equation of Weiss et al. [18] according to the following formula: NFC = 100 − (CP + EE + ash + NDFap).

To estimate daily fecal excretion, indigestible acid detergent fiber (iADF) was used as an internal indicator. The iADF was determined in the feed samples, refusals and feces, which were incubated (in situ method) for 264 h as described by Casali et al. [21] and ADF was determined after in situ incubations. The total digestible nutrients (TDN) of the diets were calculated using the nutrient digestibility according to the formula proposed by NRC [22]: TDN = dCP + dNFC + ((dEE − 1) × 2.25) + dNDF − 7, where dCP is digestible crude protein; dNFC is digestible non-fiber carbohydrates; dEE is digestible ether extract; and dNDF is digestible NDF.

Milk production was recorded with the use of meters (Waikato MKV) coupled to milking equipment. For statistical analysis, only the data referring to the collection periods were used. On the 15th and 16th days, milk samples were collected and proportionally composited based on milk yield during the morning and afternoon milking. The samples were stored in flasks containing Bromopol^®^ (2-bromo-2-nitropopan-1,3-diol) and analyzed for fat, protein, lactose, casein, milk urea nitrogen (MUN) and total solids content by infrared spectroscopy (model Bentley 2000; Bentley Instrument Inc., Chaska, MN) [23]. The daily energy secretion in milk was calculated using the NRC [22] equation; energy-corrected milk production (ECMP) was calculated according to Dias Júnior et al. [24]: ECMP = Daily milk energy secretion/0.7. The efficiency of milk production was calculated by dividing the ECMP by the DM intake.

Analysis of conjugated diene hydroperoxides in milk was performed as described by Kiokias et al. [25]. An aliquot of 50 μL of milk and 2.5 mL of isooctane/2-propanol solution (2:1 v/v) were homogenized by vortex for one minute. The solution was filtered on a 0.22 μm PTFE membrane and the filtrate was read at an absorbance at 232 nm in a spectrophotometer (UV-M51; Bel Photonics, Piracicaba, SP). The amount of conjugated diene was expressed in mmol/kg fat.

Analysis of thiobarbituric acid reactive substances (TBARS) was performed as described by Vyncke [26] with adaptations. An aliquot of 500 μL of milk was mixed with 2 mL of thiobarbituric acid (1:99 w/v), trichloroacetic acid (15:85 w/v) and hydrochloric acid (0.005:99.995 v/v), and the mixture was heated at 100 °C for 15 min. Sequentially, the mixture was subjected to a 5 min cold bath and centrifugation of 3000 *g* for 10 min. The optical density of the supernatant fluid was read at 538 nm in a spectrophotometer. The TBARS values were expressed in mmol of malondialdehyde (MDA) per kg of fat.

The milk reducing power was analyzed as described by Zhu et al. [27] with modifications. We added 2.5 mL of trichloroacetic acid (20:80 w/v) to 2.5 mL of milk; the solution was stirred by vortex for 10 min and subsequently centrifuged for 10 min at 3000 *g*. An aliquot of 1 mL of the supernatant fluid was mixed with 2.5 mL of phosphate buffer (50 mmol/L, pH 7) and 2.5 mL of potassium ferricyanide (1:99 w/v) and incubated at 50 °C for 20 min. Sequentially, 2.5 mL of trichloroacetic acid (10:90 w/v) was added and the mixture was again centrifuged at 3000 *g* for 10 min. The supernatant (2 mL) was recovered and mixed with 0.5 mL of ferric chloride (0.1:99.9 w/v) at the time of reading at absorbance at 700 nm. The reducing power was expressed in gallic acid equivalent (GAE, mg/L).

For the microbial synthesis estimates, milk samples for the analysis of allantoin were deproteinized using 5 mL of trichloroacetic acid, filtered through a qualitative filter paper and analyzed for the concentration of allantoin by the method of Chen and Gomes [28]. Spot urine samples were collected at approximately 4 h after feeding the morning on the 17th day of each experimental period. An aliquot of 10 mL of urine was diluted in 40 mL of sulfuric acid (0.036 N) and intended for the quantification of creatinine concentrations by the end point colorimetric method, uric acid by the enzymatic colorimetric method and allantoin by the colorimetric method according to the same methodology used for milk. The daily creatinine excretion was 24.05 mg/kg body weight according to Chizzotti et al. [29]. Microbial synthesis was calculated according to the formula proposed by Chen and Gomes [28]: Y= (70X)/(0.83 × 0.116 × 1000) where 70 is the N content of purines; 0.83 is the digestibility of microbial purines; and 0.116 is the ratio of the bacterial N-purine to total N. The absorption of microbial purines was estimated by the equation proposed by Verbic et al. [30]: Y= 0.85X + 0.385 BW^0.75^, where 0.85 is the absorbed purines recovered and 0.385 BW^0.75^ is the endogenous contribution to purine excretion.

Blood was collected at the 20th day of each experimental period from the coccygeal vein by using vacuum tubes without anticoagulant. Samples were collected prior to feeding and four hours after feeding in the morning, and the samples were centrifuged at 3200 *g* for 15 min to obtain serum. In the samples collected before feeding the levels of blood urea nitrogen, cholesterol, triglycerides and glucose were analyzed by the enzymatic colorimetric method, creatinine and gamma glutamyltransferase (gamma GT) by the colorimetric kinetic method and alkaline phosphatase, aspartate aminotransferase (AST), alanine aminotransferase (ALT), magnesium, phosphorus and calcium by the colorimetric method, by means of an automatic calibration spectrophotometer with a high performance reader (Flexor Biochemical Analyzer EL 200; Elitech Group, Vitoria, ES). For the samples collected 4 h after feeding, only the levels of blood urea nitrogen (BUN), cholesterol, triglycerides and glucose were analyzed. Blood tests were performed using commercial standards.

For statistical analysis, the mixed procedures of SAS (Statistical Analysis System, version 9.2) was used. The statistical model used was:

γ_ijk_= μ + Τ_i_ + p_j_ + a_k_ + ɛ_ijk_, (1)

where γ_ijk_ = dependent variable, μ = mean, Τ_i_ = fixed treatment effect (i = 1 to 5), p_j_ = random effect of the period (j = 1 to 5), a_k_ = random effect of the animal (k = 1 to 5), and ɛ_ijk_ = residual error.

Data normality was tested through the Shapiro–Wilk Test. When there was an effect of treatment, orthogonal polynomial contrasts were used to evaluate the response pattern to the increasing levels of CT using the mixed procedure of SAS. Prediction equations were also generated for those significant variables using the regression procedure of SAS. Significance was declared at *p* ≤ 0.05 and trend when 0.05 < *p* ≤ 0.10.

## 3. Results

Inclusion of CT in the diet did not influence the intakes of DM, OM, CP, EE, NDF, NFC, and TDN of lactating cows (Table 3) (*p* > 0.10). CT inclusion up to 15 g/kg of DM quadratically increased digestibility of DM and NDF (*p* < 0.05) (Table 4) and maximum values were estimated at 12.2, 11.4, CT in the diet DM, with an increase by a maximum of 7.65% and 12.85%, respectively. The OM digestibility tended to quadratically increase with added CT up to 15 g/kg of DM (*p* < 0.10). The digestibility of CP, EE, and NFC were not affected (*p* > 0.10) by the treatments. The TDN content of the diets increased linearly (*p* < 0.05) with the inclusion of CT.

Excretion of allantoin, uric acid, total and absorbed purines, daily production of microbial protein and the efficiency of microbial synthesis (g microbial CP produced per kg of TDN ingested) were not influenced (*p* > 0.10) by the inclusion of CT (Table 5).

Regarding blood parameters (Table 6), the concentrations of AST, gamma GT, calcium, phosphorus, magnesium and cholesterol before feeding and triglycerides, glucose and BUN before feeding and four hours after feeding were not influenced (*p* > 0.10) by CT levels. ALT concentrations increased linearly (*p* = 0.04), being 17.55% higher at the maximum inclusion of CT compared to the control treatment. Cholesterol at four hours after feeding had a tendency to increase linearly with the levels of CT (*p* <0.10).

Milk production, energy-corrected milk yield, fat, lactose, total solids and milk urea nitrogen did not differ (*p* > 0.10) with increasing CT levels (Table 7). Energy corrected milk and casein was reduced linearly (*p* < 0.05) with inclusion of this additive, with a reduction in casein concentration in milk (g/kg) of 7.76% with the maximum inclusion of CT compared to the control treatment. There was a trend for a reduction in milk protein content (*p* < 0.10) with a reduction of 7.11% in the treatment with greatest CT inclusion.

In relation to the milk oxidative profile (Table 8), the conjugated diene concentrations increased linearly (*p* < 0.03) with the level of CT, whereas the TBARS and the reducing power were not influenced by the CT levels (*p* > 0.10).

## 4. Discussion

CT used at high concentrations usually have a negative effect on feed intake, however this effect may vary with tannin source [31]. Another factor that could have caused the reduction in DMI in this study was the relatively low levels of protein in the diets [32]; however, even with these CP levels in the diet, the inclusion of CT did not influence the intake of DM and nutrients.

Aguerre et al. [33] used a mixture of condensed and hydrolysable tannins extracted from quebracho (*Schinopsis* spp.) and chestnut (*Castanea sativa*) extract at inclusion levels of 0, 4.5, 9 and 18 g per kg of diet DM and observed a linear reduction in DM intake, reaching an 8.2% reduction, demonstrating that the source of tannins is one of possible explanations for the different results.

The digestibility of DM, OM and NDF changed quadratically to tannin levels with maximum values estimated at 12.2, 11.9 and 11.4 g of CT per kg of DM, respectively. The reduction in NDF digestibility when tannin levels exceeded 12 g/kg dietary DM may be related to the action of CT in the formation of complexes with cell wall carbohydrate, which reduces the adhesion of microorganisms, or directly affects cellulolytic microorganisms, which negatively impacts fiber degradation [34], or by a reduction in ammonia concentration in the rumen [35] and interactions of tannins in the intestine [8].

The reduction in digestibility with the highest CT levels is similar to the findings of Aguerre et al. [33] who evaluated the use of CT extracted from Quebracho and chestnut with CT levels up to 18 g/kg DM for lactating cows and obtained a reduction in the digestibility of DM, OM, CP and NDF.

The absence of effect on the digestibility of CP and NFC may be indicative that the ruminal degradation of these nutrients has not changed substantially with the inclusion of CT, since the microbial protein synthesis, though reduced by over 8%, was not significantly depressed by CT addition. Grainger et al. [16] used CT extracted from *Acacia mearnsii* in lactating cows’ diets with levels up to 19.1 g/kg DM, however, they observed a reduction of 29% in the N digestibility. This difference in results at similar doses may have occurred because of the drench supply to the animals in the study of Grainger et al. [16], which may have been more reactive than in the present study, where the product was mixed with the concentrate.

CT can form complexes with proteins and carbohydrates [34] reducing the nutrient supply for protein synthesis in the rumen, however, in the present study the microbial synthesis was not altered, indicating that there was no limitation in the release of nutrients.

Regarding blood parameters, glucose, BUN and triglyceride concentrations were not altered with the inclusion of CT, possibly due to the absence of effects on feed intake and the digestibility of most nutrients.

AST, ALT and gamma GT are important catabolic enzymes that play an important role in the hepatic function of animals [36], and their increased activity is often related to disturbances in the functioning of this organ [37]. The inclusion of CT in up to 20 g/kg of DM did not interfere with the AST and gamma GT, but increased blood concentrations of ALT, potentially demonstrating an effect on the hepatic activity of animals. In contrast, Dei and De [38] evaluated CT of *Ficus bengalensis*, with dried and ground leaves, reaching a CT dose of 15 g/kg of diet DM for lactating cows and did not obtain differences in the concentrations of AST and ALT. According to Cozzi et al. [39] ALT values in the range of 25 to 46 IU/L can be considered normal for Holstein lactating cows. However, Du et al. [40] considered ALT values below 35.8 IU/L as indicators of liver damage for cows with ketosis.

Besides complexing with proteins, tannins and other polyphenols can also bind to some minerals [41], however, blood concentrations of calcium, phosphorus and magnesium were not altered by the inclusion of CT. It would have been interesting to assay fecal mineral samples to estimate apparent mineral absorption, unfortunately we did not have the capabilities to do so for this trial. The effects of CT on reducing mineral absorption should not be disregarded. Naumann et al. [3] pointed out that there is great variability of chelation effects between tannins and minerals and how this effect can reduce mineral availability. Waghorn et al. [41] evaluated the use of CT in *Lotus pedunculatus* (55 g/kg of DM) for sheep and reported that CT reduced sulfur absorption and increased net absorption of phosphorus and zinc, whereas effects on other minerals (Fe, Cu, Ca, P and Mg) were not altered.

Milk production and ECMP were not influenced by CT levels, however, energy-corrected milk production was reduced linearly as a result of the reduction in milk protein concentration. It was expected that the inclusion of a certain level of CT would increase the intestinal protein supply, without affecting the microbial synthesis and consequently increase the efficiency of production, however, this apparently did not occur. Studies that evaluated the use of CT in the diet of lactating cows have also showed no effect on milk yield [4,31,33], while others have reported a reduction in this variable [16,17]. It is important to note that TDN values assign protein a value of 4 kcal/g, whereas absorbed amino acids available for transfer to milk have 5.7 kcal combustible energy/g. As a result, protein in milk or retained in body tissues has more energy than TDN values would indicate. This is particularly important for determining the “true” efficiency of digested energy use. In the context of this study, because tannins increased digestibility while decreasing milk protein production, this may suggest that feeding tannins might have greater value for cows during the dry period rather than during lactation.

In the present study, the concentration of fat in milk was not influenced by CT levels; this result is similar to the results observed by others who reported that different sources of tannins did not influence milk fat concentration. Grainger et al. [16] observed a reduction in milk fat content with the use of CT, with a reduction in digestible energy.

The absence of a significant effect on ruminal microbial protein synthesis and on apparent total tract digestibility of CP indicate that the protein supply for the animal was not affected, so the reduction of protein and milk casein may have occurred due to greater intestinal protein demand and lesser supply of this nutrient to the mammary gland. CT may increase endogenous protein loss due to the interaction of these compounds with intestinal mucosal proteins, therefore, this effect could result in a compensatory use of metabolizable protein for the protection of intestinal epithelium [34,42]. Also, the relatively low values of CP of diets in this study may have contributed to the reduction in the concentration of milk protein. Beauchemin et al. [43] suggested a complete dissociation of CT-protein complexes in the abomasum may not occur, lowering the digestion of crude protein in the total tract [43] or CT may bind with other proteins or digestive enzymes in the small intestine [44].

Aprianita et al. [45] used CT extract from *A. mearnsii* and found alterations in the metabolism of protein and carbohydrates, which resulted in a reduction in protein and casein production in milk, the authors attributed their results to less microbial protein synthesis in the rumen. Similarly, Koenig et al. [46] evaluated CT from *Acacia mearnsii* at 2.5% of DM for steers and observed a reduction in apparent total tract digestion of protein, indicating that the complexation of CT and feed protein persisted throughout the gastrointestinal tract. They also indicated that reduced microbial degradation of dietary protein, host enzyme digestion, and intestinal absorption of amino acids may also have been inhibited.

The concentration of TBARS and the reducing power observed in this study were not influenced by tannins inclusion. Molecules with high molecular weight, such as tannins are not absorbed, and its action may be due to indirect effects on metabolism [13]. Luciano et al. [47] reported that the use of quebracho tannin extract at 89 g/kg of DM improved the antioxidant status of lamb muscle, while Liu et al. [48] observed a reduction in the levels of malondialdehyde in the milk of cows fed tannin extract (*Castanea sativa*) at a dose of 10 g/kg of DM (14% of CT).

Inclusion of CT increased the concentration of conjugated diene in milk indicating greater lipid peroxidation. There are reports in the literature that tannins may inhibit the last step of ruminal biohydrogenation [49] and therefore could increase the content of unsaturated fatty acids in milk [50]. According to Purba et al. [51] tannins could improve CLA production via the inhibition of C18:0 (SA: stearic acid), increasing vaccenic acid and Δ9 desaturation, which can lead to the formation of CLA in the mammary gland.

A higher content of unsaturated fatty acids in milk makes this product more susceptible to lipid peroxidation [52], since this process is characterized by the reaction between peroxidic radicals and lipids, which triggers the synthesis of conjugated dienes [53].

## 5. Conclusions

The use of CT from *Acacia mearnsii* in the diet of lactating cows increased DM digestibility in a quadratic manner, with the highest values estimated at an inclusion level of 12.2 g/kg; however, it adversely affected the animals’ performance by reducing the milk protein concentration. Therefore, based on these findings, the use of CT from *Acacia mearnsii* would be recommended for dry animals, or perhaps for growing animals due to its negative effects on milk protein concentration.

## Figures and Tables

**Table 1:** Dietary ingredients chemical composition (dry matter (DM), g/kg).

Composition	Corn Silage	Tifton 85 Hay	Ground Corn	Soybean Meal
^1^DM	276.4	856.0	868.7	879.7
^2^OM	943.5	929.3	986.2	928.3
^3^CP	77.0	89.7	91.7	473.9
^4^EE	30.5	15.2	42.0	15.0
^5^NDF	489.8	780.4	116.9	154.6
^6^NDFap	469.2	729.8	105.4	122.9
^7^NFC^1^	366.8	94.6	747.1	316.5

^1^DM—Dry matter; ^2^OM—organic matter; ^3^CP—crude protein; ^4^EE—ether extract; ^5^NDF—neutral detergent fiber; ^6^NDFap—neutral detergent fiber corrected for ash and protein. ^7^NFC—non-fiber carbohydrates calculated according to the formula proposed by Weiss [18]: NFC = 100 − (CP + EE + ash + NDFap).

**Table 2:** Ingredients proportions and chemical composition of experimental diets (DM, g/kg).

Ingredients (DM, g/kg)	Levels of Condensed Tannins Inclusion (DM, g/kg)				
	0	5	10	15	20
Corn silage	300.0	300.0	300.0	300.0	300.0
Tifton 85 hay	200.0	200.0	200.0	200.0	200.0
Ground corn	333.3	325.6	318.7	311.1	303.8
Soybean meal	146.3	147.6	148.5	149.8	151.0
Mineral mix^1^	14.8	14.8	14.8	14.8	14.8
Dicalcium phosphate	2.17	2.39	2.38	2.43	2.39
Sodium bicarbonate	3.48	3.48	3.46	3.47	3.47
Condensed tannin extract^2^	-	6.12	12.25	18.42	24.58
Chemical composition^11^					
DM^3^	692.2	693.0	693.5	693.5	694.0
OM, g/kg of DM^4^	938.6	937.6	937.1	937.2	936.8
CP, g/kg of DM^5^	138.0	139.0	140.5	140.9	140.3
RDP estimated, g/kg of CP^6^	88.2	89.4	90.6	91.6	91.0
RUP estimated, g/kg of CP^6^	49.8	49.7	50.0	49.3	49.2
EE, g/kg of DM^7^	24.6	25.7	25.3	24.5	25.9
NDF, g/kg of DM^8^	363.4	365.5	366.0	366.0	366.5
NDFap, /kg of DM^9^	337.5	337.7	336.9	336.5	336.7
NFC, /kg of DM^10^	438.5	435.3	434.4	435.4	434.0
TDN /kg of DM^6^	710.0	700.0	700.0	690.0	680.0

^1^Mineral mix—Ca: 230 g/kg; P: 50 g/kg; Fe: 500 mg/kg; Cu: 235 mg/kg; Mn: 1000 mg/kg; Zn:1875 mg/kg; Co: 30 mg/kg; I: 30 mg/kg; Se: 12 mg/kg; Na: 55 g/kg; F: 500 mg/kg; Cr: 10 mg/kg; Mg: 8 g/kg; Vitamin A—300.000 IU/kg; Vitamin D3—60.000 IU/kg; Vitamin E—400 UI/kg. ^2^Concentration of the commercial product—805.2 g/kg of CT in the dry matter. ^3^DM—Dry matter; ^4^OM—organic matter; ^5^CP—crude protein; ^6^Values estimated with the NRC (2001) models. ^6^TDN—Total digestible nutrients; ^7^EE—ether extract; ^8^NDF—neutral detergent fiber; ^9^NDFap—Neutral detergent fiber corrected for ash and protein. ^10^NFC—non-fiber carbohydrates calculated according to the proposed by Weiss [18]: NFC = 100 − (CP + EE + ash + NDFap); ^11^Analyzed chemical composition unless when specified.

**Table 3:** Intake of dry matter and nutrients of Holstein cows fed diets with five levels of condensed tannins from *Acacia mearnsii.*

Variables	Condensed Tannins Inclusion (g/kg of DM)					SEM^8^	*p* Value		
	0	5	10	15	20		CT^9^	L^10^	Q^11^
DMI (kg/d)^1^	21.6	21.0	20.7	20.1	20.0	0.70	0.12	-	-
DMI (g/kg BW)^1^	34.0	33.2	33.6	31.4	31.7	1.23	0.18	-	-
OMI (kg/d)^2^	20.2	19.7	19.4	18.5	18.8	0.65	0.11	-	-
CPI (kg/d)^3^	2.9	2.9	2.9	2.8	2.8	0.11	0.30	-	-
EEI (kg/d)^4^	0.5	0.5	0.5	0.5	0.5	0.03	0.49	-	-
NDFI (kg/d)^5^	7.8	7.6	7.4	7.1	7.3	0.31	0.24	-	-
NDFI (g/kg BW)^5^	12.3	11.9	12.0	11.3	11.5	0.55	0.39	-	-
NFCI (kg/d)^6^	9.5	9.2	9.1	8.7	8.7	0.29	0.18	-	-
TDNI (kg/d)^7^	14.1	14.4	14.4	13.9	14.0	0.55	0.81	-	-

^1^DMI—Dry matter intake; BW – Body Weight; ^2^OMI—Organic matter intake; ^3^CPI—Crude protein intake; ^4^EEI—Ether extract intake; ^5^NDFI—Neutral detergent fiber intake; ^6^NFCI—Non-fiber carbohydrate intake; ^7^TDNI—Total digestible nutrients intake; ^8^SEM—Standard error of mean; ^9^CT—effect of treatment; ^10^L—Linear; ^11^Q—Quadratic.

**Table 4:** Apparent digestibility of dry matter and nutrients (g/kg) of Holstein cows fed diets with five levels of condensed tannins from *Acacia mearnsii.*

Variables	Condensed tannins inclusion (g/kg of DM)					SEM^8^	*p* Value		
	0	5	10	15	20		CT^9^	L^10^	Q^11^
DMD^1^	633	658	675	681	656	13.75	0.03	0.04	0.01
OMD^2^	663	684	697	706	679	13.81	0.07	-	-
CPD^3^	656	667	674	660	647	17.35	0.11	-	-
EED^4^	725	750	775	783	766	21.54	0.59	-	-
NDFD^5^	436	477	488	488	460	14.13	0.01	0.08	0.01
NFCD^6^	812	825	835	853	827	15.71	0.30	-	-
TDN^7^	655	683	698	704	698	15.42	0.03	0.01	0.08

^1^DMD—Dry matter digestibility; ^2^OMD—Organic matter digestibility; ^3^CPD—Crude protein digestibility; ^4^EED—Ether extract digestibility; ^5^NDFD—Neutral detergent fiber digestibility; ^6^NFCD—Non fiber carbohydrate digestibility; ^7^TDN—Total digestible Nutrients: TDN= dCP + dNFC + ((dEE − 1)*2.25) + dNDF − 7; ^8^SEM—Standard error of mean; ^9^CT—Effect of treatment; ^10^L—Linear; ^11^Q—quadratic; ^1^Ŷ = 630.582 + 78.208x − 32.168x^2^; ^5^Ŷ = 436.492 + 96.849x − 42.456x^2^; ^7^Ŷ = 671.492 + 16.346x.

**Table 5:** Ruminal microbial protein synthesis of Holstein cows fed diets with five levels of condensed tannins from *Acacia mearnsii.*

Variables	Condensed tannins inclusion (g/kg of DM)					SEM^3^	*p* Value		
	0	5	10	15	20		CT^4^	L^5^	Q^6^
	Excretions (mmol/day)								
Alantoin	489	450	467	467	443	23.22	0.36	-	-
Uric acid	36	40	39	40	33	2.58	0.10	-	-
Total purines	526	489	506	507	486	24.98	0.42	-	-
	Microbial purines (mmol/day)								
AbsP^1^	468	432	449	450	430	24.91	0.43	-	-
	Microbial Production (g/day)								
Micr. CP, g/day	2128	1963	2041	2045	1954	113.19	0.43	-	-
Micr. Effic.^2^	153	139	142	148	142	10.99	0.64	-	-

^1^AbsP—absorbed purines; ^2^Micr. Effic.—Microbial synthesis efficiency = g of microbial CP/kg of ingested TDN; ^3^SEM—standard error of mean; ^4^CT—treatment effect; ^5^L—linear; ^6^Q—quadratic.

**Table 6:** Blood parameters (mg/dL) of Holstein cows fed diets with five levels of condensed tannins from *Acacia mearnsii.*

Variables	Condensed tannins inclusion (g/kg of DM)					SEM^5^	*p* Value		
	0	5	10	15	20		CT^6^	L^7^	Q^8^
Before feeding									
BUN^1^	15.92	17.73	18.60	17.30	17.46	1.29	0.39	-	-
Cholesterol	135.6	124.8	145.2	130.8	147.2	8.35	0.91	-	-
Triglycerides	7.80	8.00	9.40	10.4	8.80	1.01	0.13	-	-
Glucose	66.0	71.4	68.2	70.8	68.40	3.57	0.62	-	-
Creatinine	94.5	86.1	92.5	106.1	95.34	10.69	0.49	-	-
AST (IU/L)^2^	70.4	68.8	74.0	71.6	68.00	4.37	0.68	-	-
ALT (IU/L)^3^	27.7	26.0	32.7	31.1	32.56	2.31	0.04	0.01	-
GGT (IU/L)^4^	20.2	23.4	21.0	23.2	20.20	1.99	0.48	-	-
Calcium	9.32	9.47	9.38	9.16	9.40	0.32	0.89	-	-
Phosphorus	6.26	6.63	6.80	7.11	6.76	0.65	0.77	-	-
Magnesium	1.74	2.03	1.96	1.76	1.99	0.10	0.03	-	-
4 h after feeding									
BUN	18.20	19.07	19.89	17.73	16.80	1.14	0.13	-	-
Cholesterol	132	115	136	138	144	9.57	0.10	0.06	-
Triglycerides	5.80	9.20	9.40	8.60	6.80	1.87	0.28	-	-
Glucose	60.00	57.20	63.00	63.00	60.40	2.29	0.13	-	-

^1^BUN—Blood urea nitrogen; ^2^AST—Aspartate aminotransferase; ^3^ALT—Alanine aminotransferase; ^4^GGT—Gamma glutamyl transferase; ^5^SEM—standard error of mean; ^6^CT—treatment effect; ^7^L—linear; ^8^Q—quadratic; ^3^Ŷ = 27.053 + 2.974x.

**Table 7:** Milk production and composition from Holstein cows fed diets with five levels of condensed tannins from *Acacia mearnsii.*

Variables	Condensed Tannins Inclusion (g/kg of DM)					SEM^8^	*p* Value		
	0	5	10	15	20		CT^9^	L^10^	Q^11^
MP (kg/d)^1^	28.63	28.57	29.07	27.90	27.90	1.03	0.75	-	-
ECMP (kg/d)^2^	26.59	26.58	27.15	24.82	24.43	0.85	0.03	0.01	-
ECMP/DMI^3^	1.26	1.28	1.31	1.27	1.23	0.05	0.68	-	-
Fat (g/kg)	32.73	32.42	33.24	30.62	30.18	1.33	0.15	-	-
Protein (g/kg)^4^	31.07	31.69	30.72	29.42	28.86	0.93	0.05	0.01	-
Lactose (g/kg)	46.03	45.23	46.30	45.04	45.23	1.09	0.51	-	-
Casein (g/kg)^5^	24.36	24.90	24.20	22.79	22.47	0.81	0.05	0.01	-
TS (g/kg)^6^	116.7	117.7	121.8	114.2	114.1	3.30	0.19	-	-
MUN (mg/dL)^7^	14.91	16.27	16.09	15.29	14.92	0.65	0.16	-	-

MP—Milk production; ^2^ECMP—Energy corrected milk production = MP × (0.0929 × %fat + 0.0547 × %protein + 0.0395 × % lactose)/0.7; ^3^ECMP/DMI—Efficiency of milk production corrected to energy; TS—Total solids; MUN—Milk urea nitrogen; ^8^SEM—standard error of mean; ^9^CT—treatment effect; ^10^L—linear; ^11^Q—quadratic; ^2^Ŷ = 27.126 – 1.213x; ^4^Ŷ = 31.690 – 1.338; ^5^Ŷ = 24.922 – 1.178x.

**Table 8:** Oxidative profile of milk from Holstein cows fed diets with five levels of condensed tannins from *Acacia mearnsii.*

Variables	Condensed Tannins Inclusion (g/kg of DM)					SEM^4^	*p* Value		
	0	5	10	15	20		CT^5^	L^6^	Q^7^
CD^1^	39.8	37.2	40.9	40.5	46.4	2.41	0.03	0.01	0.07
TBARS^2^	1.93	1.63	1.46	1.92	1.85	0.22	0.21	-	-
Reducing power^3^	22.2	19.1	22.2	18.2	16.6	3.15	0.34	-	-

^1^Conjugated dienes (mmol/kg of fat); ^2^TBARS—Thiobarbituric acid reactive substances (mmol of malondialdehyde/kg of fat); ^3^Reducing power—(mg of gallic acid equivalent/L); ^4^SEM—standard error of mean; ^5^CT—treatment effect; ^6^L—linear; ^7^Q—quadratic; ^1^Ŷ = 37.675 + 3.302x.
